# Cleaning Up or Throwing out the Psychological Insight with the Bath Water

**DOI:** 10.1007/s12124-020-09573-w

**Published:** 2020-08-18

**Authors:** Line Joranger

**Affiliations:** Department of Health, Social and Welfare Studies, University of South-Eastern Norway, Kjølnes Ring 56, 3918 Porsgrunn, Norway

**Keywords:** Concepts, Meaning, Expectations, Meta-theory, Cognitive factors, Culture, Critical question

## Abstract

In their article *Psychology: a Giant with Feet of Clay*, Zagaria, Andò and Zennaro aim to clean up the confusing and inconsistent conceptual landscape in current psychology. They find that evolutionary psychology with its dialectical focus on nature and nurture could be the unifying meta-theory that contemporary psychology is depending on in order to compete with harder sciences, such as biology and physiology. The aim of developing a unified conceptual consensus in psychology is flattering. However, the view depends on a worldview that one can reach a psychological science with objective properties through universal concepts that are non-effected by cognitive factors. My point of view is that psychological concepts carry a great deal of implicit theoretical baggage because they come with rich connotations, acquired through everyday usage. My view has got a methodological point that leads to considerations concerning the question: how psychologists are to study concepts in order to understand them? To grasp the meaning of a given concept in its context means to understand not only its literal meaning but also how it can be applied to the world and what is done by it. All these dimensions of the meaning of a concept are deeply rooted in the respective diachronic and synchronic contexts, and that is why a psychologist should be radically prepared to change her or his expectations considering the meaning of any concept under study.

## Introduction

In the paper: Psychology: a Giant with Feet of Clay, Zagaria et al. (2020) point out that current psychology is characterized by unclear concepts and unsatisfactory definitions which make it difficult to find a common psychological core construction understood as a unified scientific theory and discipline. Investigating concepts and definitions in 11 popular introductory textbooks, Zagaria et al. find that there are few objectives held “core knowledge” in the psychological discipline.

Zagaria et al. (2020) stress that although current psychology must be intended as a pluralistic and dialectic approach rather than a monolithic one, a more unified conceptual consensus will cure the unsatisfactory ‘softness’ that lies in the plural and inconsistent definitions of current psychological core-concept, and make it “harder”, more resistant to cognitive factors, and competitive to “hard” sciences such as physic, chemistry and biology. Compared to the hard sciences, soft sciences seems to have less consensus in their core, a minor capacity to accumulate knowledge and a minor adherence to the data and theories, who “speak less from themselves” and are more likely to be influenced by non-cognitive factors such as the academic prestige, political and ideological beliefs and so on. There is no qualitative difference rather a “graduation” between these two “groups” (Zagaria et al. 2020).

In search for a unifying conceptual tool that can heal the conflicting theoretical landscape in current psychology to make its epistemological status become more like a paradigmatic discipline and not like a pre-paradigmatic one (cf. Kuhn 1970), Zagaria et al. (2000) suggest that evolutionary psychology with its dialectical focus on nature and nurture may represent a compelling meta-theory, which focus is directly related to “hard” sciences, such as physics and biology. The nature part of evolutions psychology represents a biophysiological focus related to natural science’s first principle focus, which posits a universal and inborn tendencies (causality) in every individual, which also are obviously shaped by the environment (nurture) in which they unfold. To Zagaria et al. sciences with a defined first principles, that is, a axioms (nature), which everything depends on, represent the paradigmatic status that “hard” sciences rely on. It is also the most compelling approach to frame the environmental influences intervening in shaping these tendencies.

The idea of cleaning up in the confusing and inconsistent conceptual landscape in current psychology is flattering. Developing a psychological core-concept with an inborn axiom or first principle that everything depends on and that everyone can agree on, represents an evidence based thinking that would make it easier to get funding’s and compete in a landscape where hard data and evidence based randomised research designs are the only data that counts. However, the view of consistency and hardness depends on a worldview that one can reach a science with objective properties through universal concepts that are non-effected by cognitive factors. If one recognises that psychology is dealing with cognitive factors connected to subjective issues unique for each individual, it will be difficult if not impossible to reach a “hard” psychology representing universal laws with a non-argumentative first principal (evolution).

Zagaria et al. (2020) find that “cognitive functions” often are the main object of study of scientific psychology. However, since there is no clear definition of the concept ‘cognition’, they fail to find a specific list of “cognitive function” and “concepts” anywhere. They make a list of cognitive functions that they call “terms” grouped under the *umbrella-term* “cognitive functions”, “which exclusive selection is no doubt arbitrary”. ‘Term’ and ‘concepts’ are used interchangeably throughout the article. To the list of cognitive factors 12 terms/concepts are presented. Among these are; ‘attention’, ‘cognition’, ‘consciousness’, ‘decision-making’, ‘language’, ‘learning’, ‘memory’, ‘perception’, ‘reasoning’, ‘thinking’. To the list of terms/concepts they also add three terms due to their importance in everyday psychology, although they are not usually considered “cognitive functions”. These are all “soft” concepts, such as ‘emotion’, ‘motivation’, and ‘sensation’.

In this paper I will argue that the heroic attempt to clean up in the chaotic theoretical and conceptual landscape in current psychology to make it harder and less effected by cognitive factors, will probably throw out the baby with the bath water. The confusing conceptual landscape that represent current psychology reveal itself in Zagaria et al.’s article in such a degree that it is impossible to think of a psychology without being affected by subjective experience, intentions, emotions and sociocultural values, that is, cognitive and environmental factors.

## Human Kind and Natural Kind

Zagaria et al. (2020) relate their findings of the unclear “soft” nature of psychological concepts to the old distinction between naturalistic (*nature*) and environmental (*nurture*) psychology, among others. Nature is what we think of as pre-wiring and is influenced by genetic inheritance and other biological factors. Nurture is generally taken as the influence of external factors after conception, e.g., the product of exposure, life experiences and learning on an individual. Ian Hacking (1995) and Kurt Danziger (1997) make the same distinction when they separate between what they call the “natural” and “human” kind. Whereas natural kinds (e.g., physical objects and biological species) are defined as something that exists independently of those studying them, that is, cognitive factors, human kind is described as defined and constituted, both intentionally and unintentionally, by the aims, methods, and practices of human agents who use their cognitive skills to interpret, understand and make meaning. Danziger (1997) claims that: “Human kinds... are not natural kinds, but neither are they mere legends. They do refer to features that are real. But it is a reality in which they themselves are heavily implicated, a reality in which they are a part” (pp. 191–192). In his view a psychology that is not affected by cognitive factors such as the researchers’ and the patient’s feelings and personal believes will miss out the fact that therapeutic research and praxis always goes in a context full of cultural and subjective values that always will affect the data in a soft way.

Based on the distinction between nurture (human kind) and nature (natural kind), one can presuppose that there are two kind of qualities. One of these qualities varies with the perspective one has or takes, while the other remains constant despite any changes in perspective. The latter qualities are the objective properties, which require that data be collected through direct observation or experiment, that empirical evidence does not rely on cognitive factors such as personal arguments, beliefs, values or feelings, and that all extraneous variables need to be controlled in order to be able to establish cause and effect. Objective properties also require an aim of being able to predict future behavior from the findings of the research and that it should be possible to replicate the research, that is, repeat it with different/the same people and/or on different occasions, in order to establish, among other things, whether or not the results are similar (cf. e.g., Carmines and Zeller 1979).

Thomas Nagel (1986) explains that we arrive at the idea of objective properties in three steps. The first step is to realize (or postulate) that our perceptions are caused by the actions of things on us, through their effects on our bodies. The second step is to realize (or postulate) that since the same properties that cause perceptions in us also have effects on other things and can exist without causing any perceptions at all, their true nature must be detachable from their perspectival appearance and need not resemble it. The final step is to form a conception of that “true nature” independently of any perspective. Nagel calls that conception the “view from nowhere”. The “view from nowhere” (natural kind concepts) can to some extent be compared to what Edmund Husserl (Husserl 2001a, b) in his early works explains as an intentional (thinking) act that can trace everything back to a small set of immediate or basic unhistorical essences or ideas, which function in the purely logical and explanatory disciplines as “axioms”, or pure universal a priori conceptual truths that describe the object in general.

The substance and quality of an absolute conceptual truth lies in the idea that in and of itself it non-vacuously explains how things are, and that various perspectival views of the world are possible. Thus, a scientific account cast in the language of the absolute conception may not only be able to explain why a person’s behavior is as it is, but also why we see this behavior in one way when viewed from one standpoint and in a different way when viewed from another. Another substance and quality of an absolute conceptual truth is that if the world came in a structure as characterized by an absolute concept and we did have access to it, we could use our knowledge of it to ground predictions (which, to the extent that our theories do track the absolute structures, will be borne out). Attempts to manipulate and control phenomena can similarly be grounded in our knowledge of these structures.

Most psychologists believe that with the notion of concept, such as ‘attention’, ‘cognition’, and ‘thinking’, they have identified one of the important classes for a scientific psychology, one of those classes that support the formulation of many inductive generalizations (Gergen 2001). Thus, for decades they have been looking for generalizations about members of this class. Despite the widespread endorsement of the natural kind assumption, my point of view is to challenges this natural kind assumption. A growing body of evidence suggests that concepts do not constitute a natural kind (Joranger 2015; Rose 1998; Taylor 1989; Smedslund 1991, 2011). Hence, the notion of concept is inappropriate, if one aims at formulating scientifically relevant inductive generalizations about the human kind and the human mind. Looking back at the nature of concepts, one can assume that concepts are not nature kind.

However, many psychologists of concepts assume that mental representations share many scientifically important properties, and the psychology of concepts is expected to describe those properties (Machery 2005). Psychologists assume thereby that concepts constitute a *natural kind*. Such a view is nicely put by the psychologist Gregory Murphy (2002) in his survey of the psychology of concepts:

The psychology of concepts cannot by itself provide a full explanation of the concepts of all the different domains that psychologists are interested in. This book will not explore the psychology of concepts of persons, musical forms, numbers, physical motions, and political systems. The details of each of these must be discovered by the specific disciplines that study them.... Nonetheless, the general processes of concept learning and representation may well be found in each of these domains. For example, I would be quite surprised if concepts of musical forms did not follow a prototype structure, did not have a preferred level of categorization, and did not show differences depending on expertise or knowledge (pp. 2-3).

Murphy’s view of concepts says essentially that *concepts constitute a natural kind*, that is, a class of entities about which many inductive generalizations can be formulated. We can call the view that concepts constitute a natural kind the *natural kind assumption*. The natural kind assumption is widely accepted by psychologists, philosophers of psychology, and more generally, cognitive scientists, sometimes explicitly, e.g., Prinz (2002) and Goldstone and Kersten (2003). Arguably, it has been part and parcel of the psychology of concepts.

Since members of natural kinds have many properties in common, natural kinds are the building blocks of scientific generalizations. Many empirical sciences aim at identifying the natural kinds in their domain in order to develop adequate empirical theories. Psychology is no exception. Psychologists, like Zagaria et al., look for objective classes of entities about which scientifically relevant generalizations can be made. They introduce new notions to pick out kinds that are believed to be natural, e.g., the notion of basic emotion, and many reject notions that turn out to pick out kinds that are not natural (Griffiths 1997).

## The Nature of Concepts

When pondering the significance of scientific concepts in investigative practice, the following two questions are especially central:

1. How should we think about the nature of concepts such that they can play a role in investigative practice?
2. How should we think about the nature of investigative practices such that concepts can play a role in them?

In their article Zagaria et al. (2020) use different concepts and semantic levels to explain the soft nature of psychology. They separate between “fundamental terms” (‘psychology’, ‘mind’, and ‘behaviour’), “core concept”, “core construction”, and “category of cognitive functions”, among others. “‘Concept’ is a vague concept” (p. 433), Wittgenstein (1978) says. Vagueness is usually taken to be “soft”, that is, a defect compared to an abstract and absolute objective ideal of exactness which has been held in high esteem and dogmatically adhered to in philosophy. There is no such absolute ideal (p. 88), according to Wittgenstein (1958). However, vague concepts can very well serve our purposes even though we may not be able to apply them unequivocally in every possible case, especially in cases we call borderline. So, even if ‘concept’ is a vague concept, i.e., even if we cannot sharply determine it by an analytical definition, it does not follow that it is useless.

The problem with the concept of ‘concept’, is not so much that it may be vague as that there have been different ways of understanding it. Understanding concepts as entities in the form.

of vessels, which has dominated contemporary philosophy, has significantly contributed to the development of certain philosophical problems, such as the problem of incommensurability, which dissolve once we adopt a different understanding of concepts, namely, understanding concepts as uses of words in their sites (Kindi 2012).

No matter how desirable an absolute conceptual truth may seem, researchers’ ability to use scientific claims to represent all and only facts about the human being and the world depends on whether these claims can be unambiguously established on the basis of evidence. Researchers test scientific claims by means of their implications, and it is an elementary principle of logic that although the implications of claims are true this need not imply the truth of the claims themselves (Longino 1990, 1996; Popper 1959, 1972; Putnam 1987). Rather, they could be related to a specific sociocultural context that more or less unconsciously pushes us to think according to certain values, whether moral, personal, social, gender-specific, political or cultural (Joranger 2019; Joranger 2015; Prilleltensky et al. 2008; Prilleltensky and Vandenbos 1989).

The arguments I want to put forward to support my view is connected to conceptual historians, such as Reinhart Koselleck (1989, 2002, 2004) and Quentin Skinner (1988). It relates to the fact that in psychology as in any other scientific field, concepts are also using as tools or as weapons in political and medical discussions. This is characteristic especially in the case of value-laden concepts related to diagnosis, health, sickness, psychopharmacology, user involvement, power, freedom etc. These concepts are used in various ways to meet the ends of the political and health care actors using them. Concepts are part of the world and the world is accordingly changed using concepts. This is a theoretical insight and leads to explanations of conceptual change and its relation to social changes and serves to dissolve the sharp distinction between theory and practise. Accordingly, to study concepts, psychological or not is to study a form of cultural, political and social change.

According to the conceptual history point of view, concepts acquire their meaning from their uses in their respective historical and sociocultural contexts. This has got a kind of methodological point that leads to considerations concerning the question: how psychologists are to study concepts in order to understand them? To grasp the meaning of a given concept in its context means to understand not only its literal meaning but also how it can be applied to the world and what is done by it. All these dimensions of the meaning of a concept are deeply rooted in the respective diachronic and synchronic contexts, and that is why psychologists should be radically prepared to change her or his expectations considering the meaning of any concept under study.

The semiotic agenda in psychology has been reinforced by work in linguistics and philosophy on discourse and implicit meanings and by debates about the ambiguous and shifting boundary between semantic meanings and pragmatic meanings (cf. e.g. Gergen 1990; Innis 2002; Lakoff 2008; Machery 2009; Peirce 1991; Ricoeur 2003; Shweder and Sullivan 1993; Smedslund 2011; Taylor 1985; Valsiner 2007; Vygotsky 1962; Wierzbicka 1996; Ludwig Wittgenstein 1968). Shweder and Sullivan (1993) suggest that:

Semantic meanings (e.g. that ‘bachelor’ means a ‘marriageable unmarried male’) are implications which are necessary, and hence unalterable and invariant, across all possible contexts of application and for all possible spears. Pragmatic meanings (e.g. that ‘John is a lion’ means that ‘John is brave’), in contrast, are implications that are dependent on the context and speaker (p. 501).

The references and quotes above show that an influential position has emerged in psychology, philosophy, linguistics and literary theory, which argues that necessary and intrinsic meanings (fixed essences) are few, difficult to locate, and perhaps even nonexistent. Even though scientists dealing with psychological issues devote a great deal of effort to making their theoretical language and knowledge clear, explicit and empirical, that is, non-subjective, the meaning in which psychological phenomena are categorized carries an enormous burden of unexamined and unquestioned socio-cultural and historical assumptions and preconceptions. Speaking from a more constructivist point of view Kenneth Gergen (2001) criticizes current scientists in psychology for treating language (including numerical language), as if it were the bearer of truth through which they inform their colleagues and their culture of the results of their observations and thought. For most psychological scientists, according to Gergen (2001), the world and the individual mind are simply out there, available for observation. Danziger (1997) submits that:

By the time explicit psychological theories are formulated, most of the theoretical work has already happened – it is embedded in the categories used to describe and classify psychological phenomena. To excavate this hidden level of theory, to make it visible, we need an analysis of the discourse from which psychological categories derive their sense (p. 8).

Rose and Abi-Rached (2013) hold a comparable view. They claim that psychological labels carry a great deal of implicit theoretical baggage because they come with rich connotations, acquired through everyday usage. They believe experimental practices in laboratory settings still fail to address adequately the fact that neither animal nor human brains exist in isolation or can be understood outside their environment and form of life. Rose and Abi-Rached (2013) state that:

Conceptions of social in social neuroscience are frequently impoverished, reducing social relations to those of interaction between individuals, and ignore decades of research from the social sciences on the social shaping and distributed character of human cognitive, affective, and volitional capacity (p. 23).

Likewise, Smedslund (2011) demonstrates that, although in the psychological field one deals with semantic primitive concepts, which cannot be defined by other concepts and are therefore necessary if one wishes be understood by others (cf. Wierzbicka 1996), these concepts are linked to concepts developed by individual feelings in open systems continually interacting with an environment.^1^ Smedslund claims that humans continuously learn from their experiences, which means that we are changing in ways partially dependent on these random events, and hence, in ways that are also unpredictable. Smedslund (2011) believes this is why “the limitations of the empirical project of psychology are becoming gradually more visible, and the merits of stronger theoretical analysis of what is given a priori are becoming more plausible” (p. 134). Still, the psychologist always tries to *understand* the client in ‘hard’ logical terms, in terms of the brain, although the meaning of what is said remains a psychological mystery (Smedslund 2013). To Smedslund (2013) “one cannot be said to understand the irrational, because the concept of understanding presupposes rationality” (pp. 93–94). Instead of using psychological concepts from the hard sciences such as biology and physiology, Smedslund (1988) suggests that one should use common sense, that is, a collective source of knowledge and rationality, when approaching the human mind.

Taking into consideration how the productive power of concepts and language is shaped and invented according to subjective experiences and a specific historical and sociocultural environment, there is reason to believe that “hard” epistemic values, such as consistency, logic and objectivity, simplicity, breadth of scope, fruitfulness, etc. are not purely “hard” after all. Their use imports political and social values into contexts of scientific judgment that can lead to biases and adverse research results (cf. e.g., Burman 2008; Feyerabend 1975; Foucault 1971, 2003; Longino 1996; Putnam 1987).

Although we are self-defining actors, our involvement with other human beings and situations also affects us. Because of this specific dialectical relationship between nature and nurture, shown in evolutionary psychology, scientists who study the human psyche, as opposed to scholars in the natural sciences, cannot describe human nature independently of own cultural practices and history. A researcher or therapist working with psychological issues will always use their concepts and descriptions to shape and interpret the person(s) under investigation and it is here that the relationship between psychological objects and historical socio-cultural practices lies. This is why Kvale (1992) suggests that an alternative psychology could be to move out of the archeology of the psyche and into the cultural landscape of the present world. This would involve facing the rootedness of human existence in specific historical and cultural situations, and being open to the insights on the human condition provided by the arts and what Brinkmann (2020) calls a “nomadic psychology”. The main topic of study would precede evolutionary explanations (cf. Brinkmann 2020) and include the linguistic and social construction of reality and the interrelations of a local context and the self in a network of relationships. This would require accepting the open, philosophical perspectival and ambiguous nature of knowledge and validating knowledge through practice:

It would involve a multi-method approach to research, including qualitative descriptions of the diversity of a person’s relation to the world and a deconstruction of texts that attempt to describe this relation. The question remains whether such changes are too radical to find their place within a psychological science with strong individualistic and rationalistic roots (Kvale 1992, p. 53).

Human consciousness is primarily sociocultural and linguistically defined and mediated by symbols. Opposed to “hard” sciences, which seek pure basic, or eternal laws, like generalizations about a small number of abstract objects or forces whose interrelations can be stated in quantitative mathematic form, “soft” sciences studying things that have meaning for meaning-imposing human beings cannot escape the involvement with the semiotic sciences (e.g., linguistics, philosophical, social and personality psychology, cultural anthropology, history, etc.). The productive power of language and concepts shows that the type of language and concepts we use to explain/understand a person’s inner feeing and consciousness cannot be fixed or related to a first principle everyone agrees on. If so we lose the sight of oneself and the persons involved (Joranger 2013, 2015).

While sociocultural contexts shape the expression of human agency, they do not create it. Sociocultural conventions and practices rely on the action of the individual within them, for their influence on the development of human psychology. This is not just to make the claim that social phenomena can be discerned and made manifest as real. The social exists as a field of possibilities and constraints structured by human agents in their practices and participations.

I think we can conclude that there are external facts, and we can say what they are. What we cannot say, because it makes no sense, is what these facts are *independent* of all conceptual choices. In the course of our evolution a good deal of what is sociocultural is incorporated into both our pre-reflexive, intentional action and the more advanced reflexive consciousness that emerges as development unfolds. We cannot therefore understand human life merely in terms of an predetermined evolutionary psychological meta-concepts (Zagaria et al. 2020), which frame representations about and respond to others, because a great deal of human action happens only insofar as the agent understands and constitutes him- or herself as an integral part of a “we”. Much of our understanding of the self, society, and world is carried in practices that consist of dialogical action.

To understand the meaning of a concept or a knowledge area, such as evolution psychology, we have to stand at the border of it, asking and philosophizing about its properties and coherence. This requires that we search for the extent to which a given explanation adds to the vocabularies of cultural and historical understanding. It also requires that we ask questions about how particular conceptions of mental life came into being, and what role they have played in cultural life. For example, is it really so that the production of a psychological episteme cannot be detached from political disciplinary power mechanisms, both because these power mechanisms make possible and bring about the production of truth, and because the production of truth itself has the power to bring the psychological episteme together in a specific worldview? Such sociocultural and historical analyses are pivotal in casting light on the function played by conceptions of the mind and subjectivity within Western culture today, while they can also open our eyes to what is good and bad in our way of thinking about what it is to be human and what kind of concepts one should deal with in order to understand a particular human being.

## Footnotes

1 The perspectives forwarded in Smedslund’s discussions of Wierzbicka’s collections of semantic primitive concepts are shared by Shweder (2013).
